# Regulatory Effect of Sea-Buckthorn Procyanidins on Oxidative Injury HUVECs

**DOI:** 10.3389/fnut.2022.850076

**Published:** 2022-05-17

**Authors:** Ximeng Lin, Michael Yuen, Tina Yuen, Hywel Yuen, Min Wang, Qiang Peng

**Affiliations:** ^1^College of Food Science and Engineering, Northwest A&F University, Yangling, China; ^2^Puredia Limited, Xining, China

**Keywords:** *Hippophae* rhamnoides, proanthocyanidins, antioxidant, cardiovascular disease, HUVECs

## Abstract

As society develops and aging populations increase, the incidence of arteriosclerosis, a seriously harmful cardiovascular disease (CVD) which mostly results from endothelial cellular oxidative damage, has continuously risen. Procyanidins from sea-buckthorn is a powerful antioxidant, although its protective effect on the cardiovascular system is not yet clearly understand. In this study, oxidative damaged HUVECs induced by palmitate acid (PA) were used as a model and the regulatory effect of procyanidins from sea-buckthorn (SBP) on HUVECs were investigated. The results showed SBP can be used for 12 h by HUVECs and had no detective cytotoxicity to them under 400 μg/L. Also, different concentrations of SBP can increase mitochondrial membrane potential and NO level and decrease LDH leakage in a dose-effect relationship, indicating SBP can improve oxidative damage. In addition, western blots and qPCR results showed SBP regulation on oxidative injured HUVECs is probably through p38MAPK/NF-κB signal pathway. This study revealed the molecular mechanism of procyanidins in decreasing endothelial oxidative damage, providing a theoretical foundation for further research on natural bioactive compounds to exert antioxidant activity in the body and prevent and improve cardiovascular diseases.

## Introduction

Sea-buckthorns are precious deciduous shrubs, belonging to *Hippophae* genus in *Elaeagnaceae*. Procyanidins, as important bioactive compounds among vitamins, carotenoids, and flavonoids (1, 2), are multimeric compounds formed by the condensation of flavanols and widely exist in many plants with lower contents (3). From previous studies on procyanidins extracted from other plants, researchers found procyanidins are functionally bioactive polymers and they have antioxidant, anticancer, cardiovascular, and cerebrovascular disease prevention effects and anti-inflammatory and immunomodulatory activities (4, 5). However, studies on procyanidins from sea-buckthorns have rarely been reported.

Vascular endothelial cells (VECs) are a monolayer of cells that continuously cover the surface of the entire vascular cavity, which constitutes the starting barrier of blood vessels, and has the function of regulating blood flow, participating in substance exchange, preventing lipid leakage, inhibiting platelet aggregation, and preventing thrombosis (6–8). As society develops, CVD has gradually become the main disease that endangers health and causes atherosclerosis. Due to the pathogenesis complexity of AS, it has attracted more research attention. The trigger of AS is structural destruction and dysfunction of vascular endothelial cells (9). Although there are many factors leading to vascular endothelial cells' injury, oxidative damage of vascular endothelial cells is considered an important causative factor and has obtained more attention. Recent studies have proved procyanidins can prevent and improve oxidative injury of vascular endothelial cells, especially oligomeric procyanidins (10, 11). Also, medical and toxicology-related experiments have shown natural procyanidins have non-carcinogenic, non-toxic, and non-teratogenic advantage compared with synthetic compounds. However, whether procyanidins can prevent oxidative injury of VECs and how their protection works in resisting oxidative damage is still unclear.

In this study, oxidative damaged HUVECs cells induced by PA were used as a model. The prevention effect of procyanidins in sea-buckthorn (SBP) on oxidative damaged HUVECs were investigated through cell viability, SBP absorption, Mitochondrial membrane potential (Δψm) determination, LDH leakage, and NO level detection. Moreover, western blot and qPCR were used to study the relative mRNA and proteins of SBP improved HUVECs oxidative injury.

## Methods

### Materials and Chemicals

Cyanthox, Procyanidins from sea-buckthorn (SBP), was provided by Puredia Limited Co. (Qinghai, China). SBP yield was 9.1% compared with sea-buckthorn powder. The purity of SBP was 91.5% compared with standard procyanidins (95%). LC-MS/MS revealed SBP mainly contained four compounds: (-)-epicatechin gallate (C_22_H_18_O_10_), procyanidin B (C_30_H_26_O_12_), (+)-gallocatechin-(+)-catechin (C_30_H_26_O_13_), and (+)-gallocatechin dimer (C_30_H_26_O_14_).The antibodies of LOX-1, ICAM-1, p-NF-κB, and p-p38 were purchased from Cell Signaling Technology (Shanghai, China). PA was provided by Sinopharm Chemical Reagent Co. (China). All other chemicals and solvents used in this study were of analytical grade.

### Cell Culture

HUVECs cells were purchased from ATCC. The cells were cultured in H-DMEM medium (Gibco Co., China) with 10% FBS (BioInd Co., Israel),100 U/mL penicillin (Erye Pharma Co., China), and 100 μg/mL streptomycin (NanJing SunShine Biotechnology Co., China) at a 37°C and in a 5% CO_2_ incubator. Between 3 and 10 passages of HUVECs were used in this study.

### Cell Viability Assay

HUVECs (5 × 10^4^ cells/mL) were cultured in a 96-well plate for 24 h and treated with different concentrations of SBP (25, 50, 100, 200, 400, 600, and 800 μg/L) for 12 h. Afterwards, the cells were added to 10 μL CCK-8 (EnoGene Co., China) and kept for 1 h. The cell viability was determined by a multifunctional enzyme marker at a wavelength of 450 nm and expressed as relative percentage of blank control.

### SBP Absorption Detection in HUVECs

HUVECs (5 × 10^4^ cells/mL) were cultured in a 96-well plate for 24 h and then treated with 100 μg/L SBP for 1, 2, 4, 8, and 12 h. The mediums were collected and centrifuged (3000 rpm/min) for 10 min. Sulfuric acid-vanillin colorimetry was used for SBP absorption detection. The results were calculated by a formula adjusted by different concentrations of standard catechins. SBP dissolved in DMSO as blank control.

### SBP Effect on HUVECs Cell Morphology

HUVECs were incubated in 6-well plate for 24 h and then treated with 25 and 100 μg/L SBP for 30 min. Afterwards, the cells were treated with 100 μmol/L PA for 24 h. The cells morphology was observed by inverted fluorescence microscope.

### ROS Level Measurement

1.0 × 10^5^/mL HUVECs were cultured in 12-well plate for 24 h. Then the cells were co-cultured with 25, 50, and 100 μg/L SBP for 12 h. Subsequently, the cells were co-incubated with 100 μmol/L PA for 12 h. Measurement of ROS level in each group was followed by ROS kit (Beijing Solarbio Science & Technology Co., China).

### Mitochondrial Membrane Potential (Δψm) Determination

Δψm was measured by JC-1 mitochondrial membrane potential detection kit (Beyotime Biotechnology Co., China). The cultured cells were continuously incubated in H-DMEM for 3 h. Afterwards, the cells were treated with 25, 50, and 100 μg/L SBP for 30 min and then incubated with 100 μmol/L PA for 6 h. Δψm was measured at 590 nm after the cells were incubated with 1 mL JC^−1^ for 20 min.

### LDH Leakage Detection

HUVECs were cultured with H-DMEM for 3 h. The cells were pre-treated with 25, 50, and 100 μg/L SBP for 30 min and then treated with 100 μmol/L PA for 12 h. LDH cytotoxicity test kit (Nanjing Jiancheng Bioengineering Institute, China) was used for LDH leakage analysis.

### Assay for NO Detection

HUVECs were divided into six groups: blank, model, 25 μg/LSBP, 100 μg/LSBP, SB, SB, and SBP. NO detection kit (Beyotime Biotechnology Co., China) was used for NO content analysis after being cultured for 12 h.

### Western Blot and qPCR Analysis

HUVECs were divided into the same six groups: blank, model, SBP (25 and 100 μg/L), SB, SB, and SBP. After being cultured for 12 h, the cells were collected for western blot and PCR analysis. The relative proteins (LOX-1, ICAM-1, p-NF-κB, and p-p38) and the relative mRNAs (LOX-1, ICAM, NF-κB, eNOS, and iNOS) were analyzed.

### Statistical Analysis

All data were triplicate determinations expressed as means ± SD and analyzed with variance (ANOVA) followed by Duncan's multiple-range test. SPSS version 26.0 was used for statistical analysis and the definition of statistical significance was *p* < 0.05.

## Results

### Cell Viability Analysis

**Figure 1:**
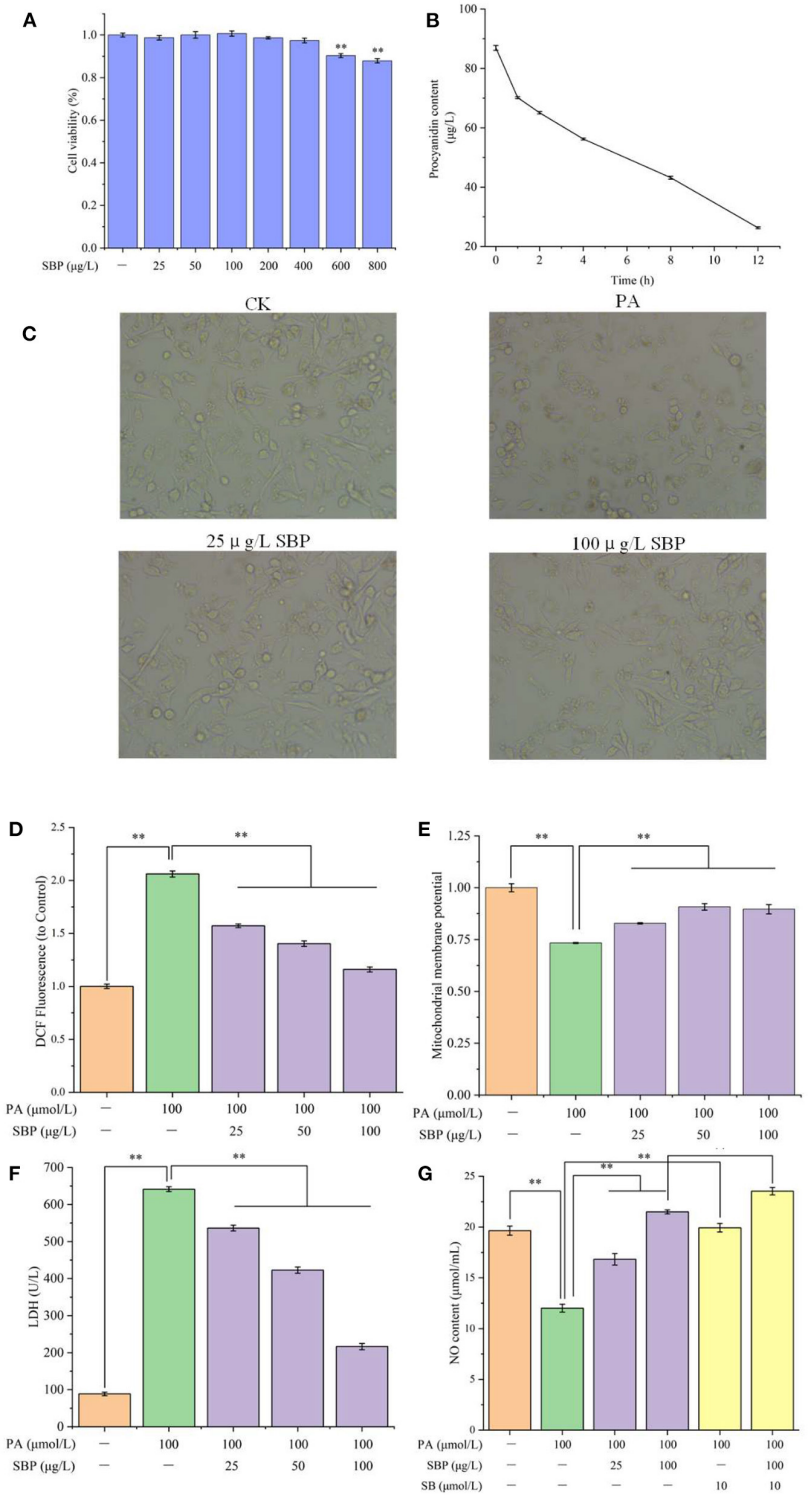
Cell viability of HUVECs with different concentrations of SBP **(A)**, SBP absorption in HUVECs **(B)**, HUVECs cellular morphology under different treatments **(C)**, Mitochondrial membrane potential of different treatments **(D)**, LDH leakage of different treatments **(E)**, NO content of different treatments **(F)**, ROS content of different treatments **(G)**. The results were expressed as the mean ± SD (*n* = 5). **p < 0.01.

The cell viability of different concentrations of SBP treatment was shown in Figure 1A. Compared with blank control, HUVECs viability did not significantly change after SBP treatment under 400 μg/L. However, when SBP treatment was above 600 μg/L, the cell viability changed extremely (*p* < 0.01). Thus, SBP concentration under 400 μg/L was selected for the following experiments.

### SBP Absorption of HUVECs Analysis

SBP absorption of HUVECs at different time points was displayed in Figure 1B. With extended time, SBP concentration in the medium decreased. After 100 μg/L SBP incubated with HUVECs for 1 h, SBP concentration had significantly decreased. Also, after co-incubation for 12 h, SBP concentration, decreasing to 26.3 μg/L, was 70% of the original concentration. The concentration of SBP dissolved in DMSO also lacked a significant difference after 12 h. This indicated SBP can gradually be absorbed and metabolized by HUVECs and did not degrade when incubated with HUVECs. Therefore, the longest time of SBP and HUVECs co-incubation is 12 h in the following experiments.

### Analysis of SBP Effect on HUVECs Cellular Morphology

Normally, HUVECs cells will adherently grow, and their morphology is of a flat cobblestone shape. After PA treatment, cellular morphology became round and their adherent growth ability was weaker. However, when pretreated with different concentrations of SBP (25 and 100 μg/L), their morphology gradually recovered. In addition, many cobblestone shape cells can be observed in the image of 100 μg/L SBP treatment group (Figure 1C).

### ROS Level Analysis

From Figure 1D, compared to blank control group, 100 μmol/L PA significantly increased ROS level in HUVECs cells, which determined PA stimulation can induce cellular oxidative stress. However, different concentrations of SBP treatment significantly decreased ROS level (*p* < 0.01) and the reduction effect increased with SBP concentration increase. This meant SBP can effectively prevent HUVECs oxidative damage induced by high level lipid.

### Δψm Determination

HUVECs oxidative damage will cause cellular mitochondria dysfunction, resulting in Δψm decrease. In Figure 1E, when HUVECs were stimulated by 100 μmol/L PA for 12 h, its Δψm significantly decreased to 0.73 compared with blank control (*p* < 0.01). Oppositely, SBP co-incubation can significantly recover HUVECs Δψm (0.90), meaning SBP can significantly prohibit Δψm decrease of HUVECs induced by PA and improve oxidative damage situation.

### LDH Leakage Analysis

LDH is a glycolytic enzyme, widely existing in the cell matrix. Once LDH leakage occurs, it means cell membranes in cells or tissues have been attacked by ROS, resulting in oxidative damage. In Figure 1F, compared with blank control (88.7 U/L), PA can extremely increase LDH level to 641.5 U/L in medium (*p* < 0.01), indicating PA-induced oxidative damage caused LDH leakage in cell matrix. However, different concentrations of SBP can reduce LDH viability in medium and have a positive dose-effect relationship. Especially, 100 μg/L SBP extremely slowed down LDH leakage of cell matrix (216.3 U/L), indicating SBP can significantly decrease HUVECs oxidative damage induced by PA, which corresponded with the results above.

### NO Content Analysis

NO, one of the most important products of HUVECs, plays a necessary role in maintaining vasodilation capacity. However, one cause of vascular disease is insufficient NO production resulting from endothelial oxidative damage. From Figure 1G, PA stimulation significantly decreased NO level (12.0 μmol/mL) while SBP pre-treatment can significantly improve NO level in HUVECs, especially the 100 μg/L SBP treatment (21.5 μmol/mL). This indicated that SBP can efficiently recover insufficient NO production in HUVECs induced by PA stimulation, resulting in improved endothelial oxidative damage and dysfunction. Compared with PA group, SB had a similar effect as SBP. However, incubation with SB and SBP did not have a superimposed effect on NO production. This meant the SBP effect on NO production is probably through p38MAPK/NF-κB signal pathway regulation.

### Relative Proteins Expression Analysis

**Figure 2:**
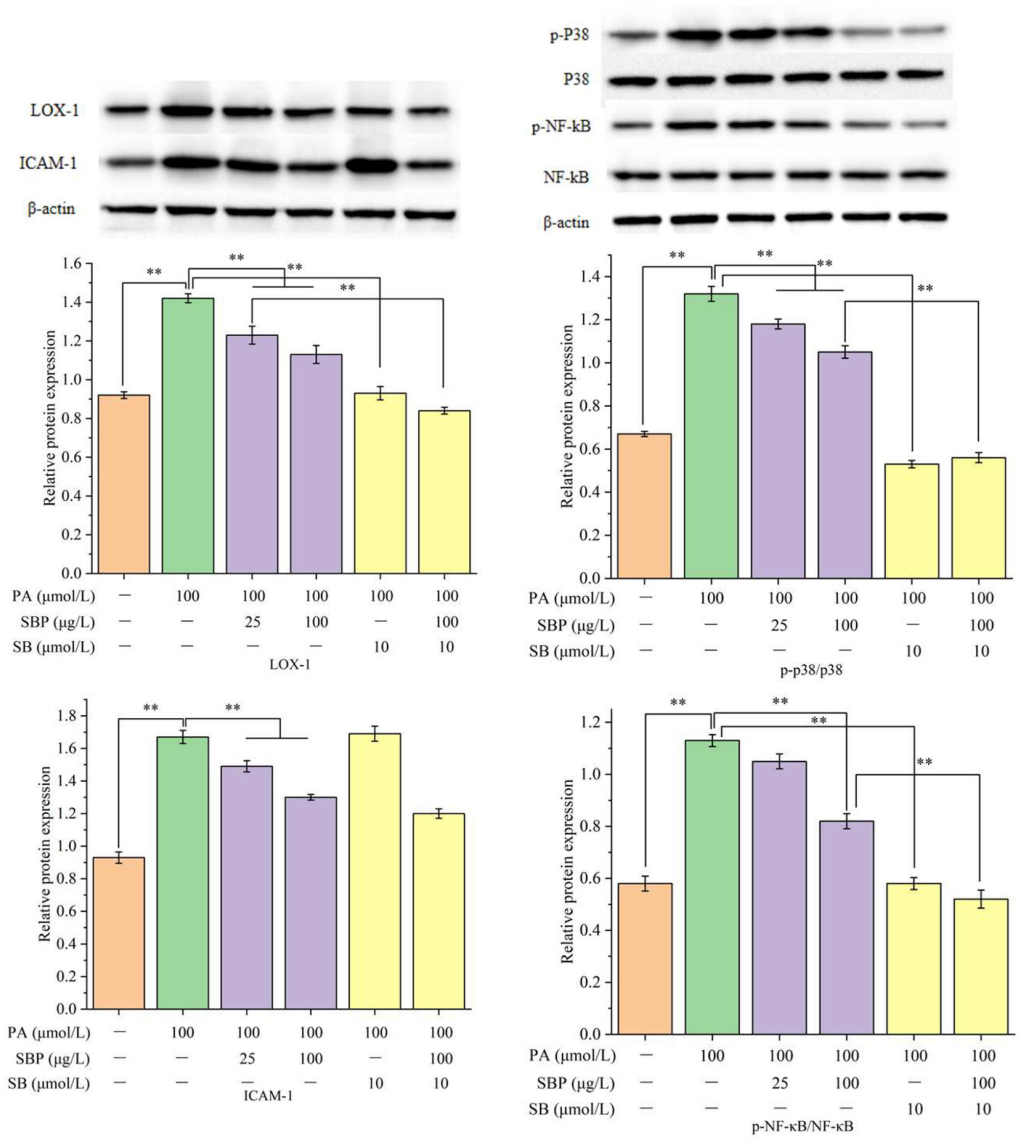
Relative proteins expression (LOX-1, ICAM-1, p-p38/p38, and pNF-κB/NF-κB) under different treatments of SBP and SB. The results were expressed as the mean ± SD (*n* = 3). **p < 0.01.

Western blots results (Figure 2) showed that PA could significantly increase LOX-1 expression while SBP and SB could slightly decrease its expression. ICAM-1 expression increased induced by PA, but higher concentration of SBP can obviously prohibit its expression, while SB had no significant effect on its expression. For p-NF-κB and p-p38 expression, PA treatment can significantly induce phosphorylation of NF-κB and p38, while higher concentrations of SBP can significantly inhibit activation of p38MAPK/NF-κB signal pathway. These results determined SBP can reduce HUVECs oxidative damage induced by PA probably through the regulation of p38MAPK/NF-κB signal pathway.

### Relative mRNA Expression Analysis

**Figure 3:**
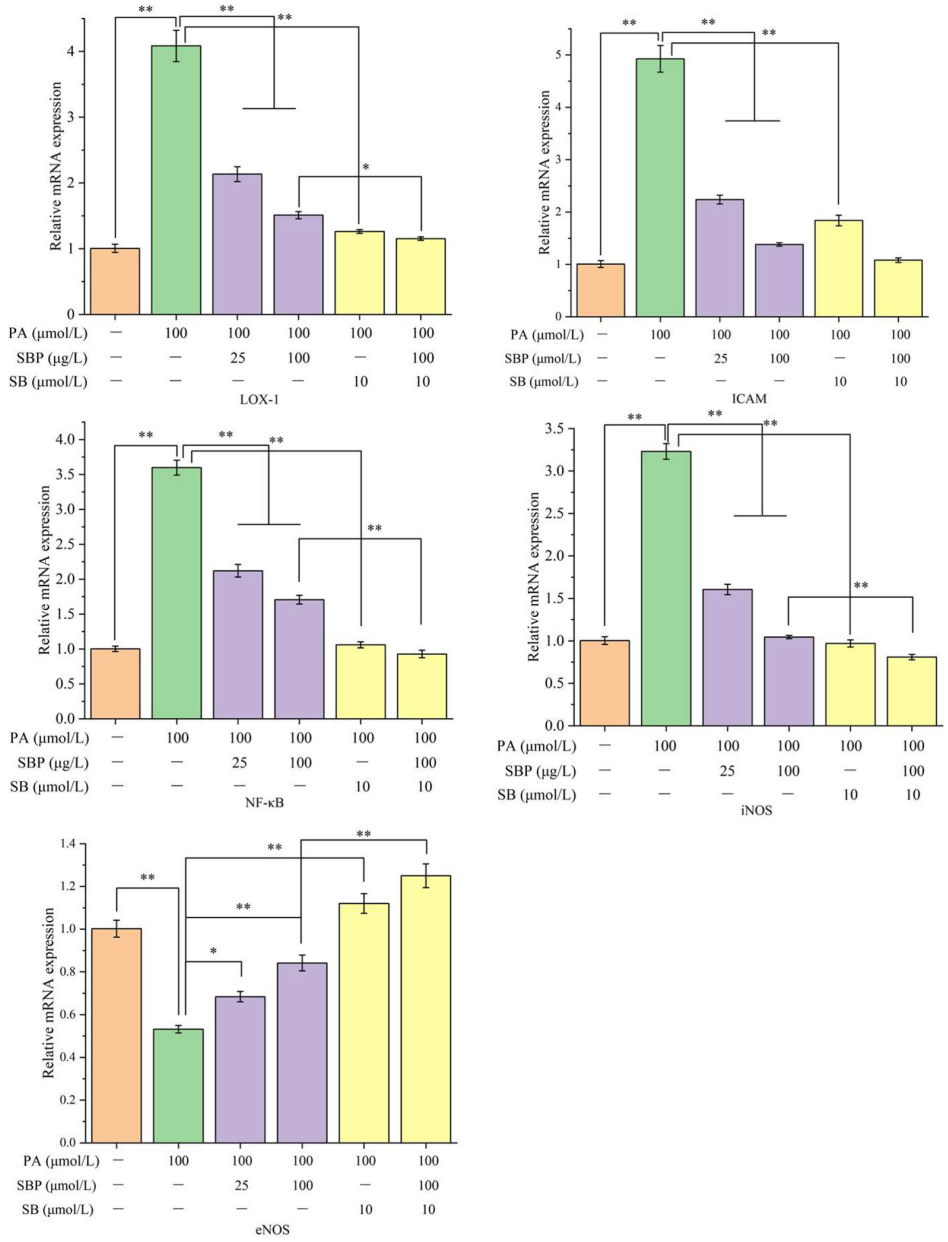
Relative mRNA expression (LOX-1, ICAM, NF-κB, iNOS, and eNOS) under different treatments of SBP and SB. The results were expressed as the mean ± SD (*n* = 3). *p < 0.05 and **p < 0.01.

Compared with blank control (Figure 3), PA stimulation significantly increased mRNAs expression of LOX-1, ICAM-1, NF-κB, and iNOS (average 3–4 times of blank control), while eNOS mRNA expression significantly decreased (about 53% of blank control). However, SBP treatment obviously inhibits this up or down regulation and has a positive does-effect relationship. Moreover, SB treatment significantly inhibited all tested mRNAs expression which indicated SBP regulation of these mRNAs expression probably has a relationship with p38MAPK/NF-κB signal pathway. This is consistent with western blot analysis.

## Discussion

Endothelial cells' dysfunction is one of the main factors of many chronic diseases, especially CVD and related complications (12). Clinical studies have shown that oxidative stress induced by high sugar or high fat leads to endothelial cells' oxidative damage, which is significantly positively correlated with endothelial cells' dysfunction and CVD (13). Therefore, efficient improvement of oxidative injury of endothelial cells can prevent and reduce the possibility of CVD. Many studies *in vitro* and *in vivo* have proved polyphenolic compounds can effectively scavenge excessive free radicals and reduce chronic diseases caused by oxidative injury, such as diabetes, cancer, and CVD (11, 14). As a polyphenolic compound, procyanidins are equipped with powerful free radical scavenging ability, which has a positive relationship with its concentration (10).

From the results above, SBP can reduce LDH leakage caused by cell membrane damage, resulting in preventing oxidative injury. Δψm is the main bioenergy parameter to measure the electron transport capacity of mitochondria, directly controlling the ATP synthesis, respiration rate, and reactive oxygen species production of cells (15). High lipid induced oxidative stress of HUVECs leads to mitochondria damage, mainly manifested as the Δψm decrease. Pretreatment with SBP can obviously recover Δψm, protect mitochondria, and improve oxidative injury. Hence, in PA induced oxidative damaged endothelial cell model, SBP revealed a great ability for improving oxidative damage. However, how SBP exerts its oxidative ability still needs to be investigated.

**Figure 4:**
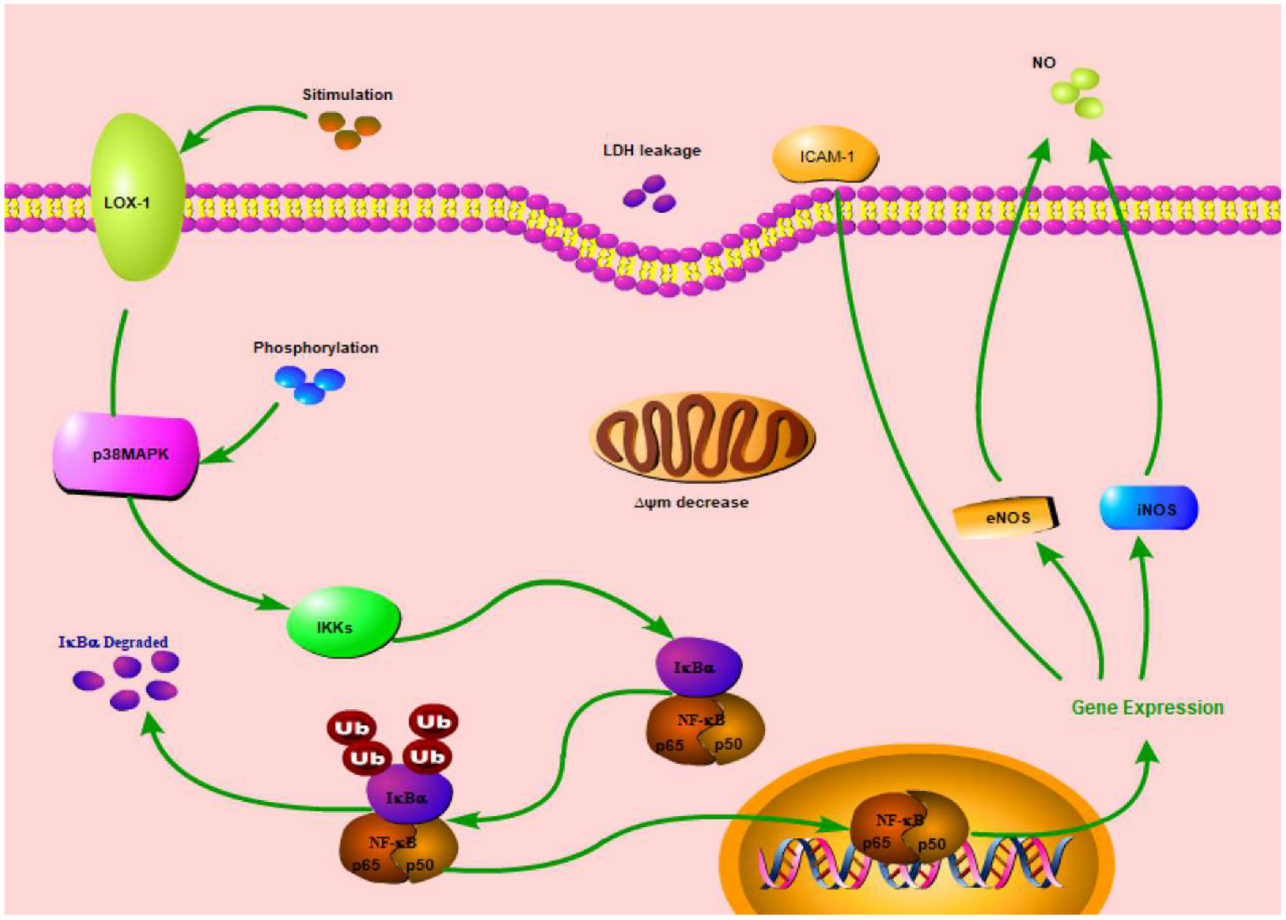
Simplified p38MAPK/NF-κB signal pathway diagram.

In this study, we also investigated SBP effect on p38MAPK/NF-κB signal pathway. The results revealed SBP effect on related indexes (LOX-1, ICAM-1, NF-κB, eNOS, and iNOS) expression was associated with p38MAPK/NF-κB signal pathway (Figure 4), but ICAM-1 protein expression had no relation with this pathway.

MAPK family is a signal for intracellular and extracellular signaling which can affect a series of biological functions during cellular growth and development. In mammalian cells, extracellular-signal regulated kinase (ERK), c-Jun N-terminal kinase (JNK), and p38MAP kinases are three MAPK family members which have been clearly characterized (16). Lipid induction can activate signal pathways in cells, such as protein kinase pathway, tyrosine protein kinase pathway, and MAPK pathways, resulting in vascular endothelial cell dysfunction or apoptosis (17). p38MAPK is a main MAPK signal pathway, closely associated with cellular inflammation and oxidative stress in HUVECs cells. In an oxidative stress situation, MKK3/6 respectively activates p38α/β and then makes p38 phosphorylate (18, 19). Afterwards, MAPK cascade reaction activates NF-κB. NF-κB is a main regulatory factor of inflammation which can regulate many genes' expression related to AS (20). In this study, SBP significantly inhibited phosphorylation of p38MAPK and NF-κB, indicating SBP probably prevents oxidative damage induced by PA through inhibition of p38MAPK/NF-κB signal pathway activation.

Endothelial cell apoptosis has been determined as a main characteristic of AS occurrence (21–23). LOX-1 expression can up-regulate the expression of pro-apoptotic factor (Bax) and down-regulate the expression of anti-apoptotic factor (Bcl-2), leading to endothelial cells' apoptosis (17). Research has proven NF-κB activation has to be regulated by LOX-1 system (24). This study showed mRNA and protein expression of LOX-1 in model group were both higher than blank control group, while its expression decreased after treatment with p38 prohibitor, indicating a protective effect of SBP on HUVECs is probably through the prevention of p38MAPK/NF-κB signal pathway activity.

NO content in endothelial cells is one important index to evaluate function of the cells (25). Oxidative stress can cause endothelial cells' dysfunction, resulting in a decrease of NO level and then insufficient diastolic capacity of blood vessels. NO is catalytically produced by nitric oxide synthase (NOS) and exerts significant function in the AS process. NOS contains neuronal NOS (nNOS), endothelial NOS (eNOS), and inducible NOS (iNOS). Normally, NO is catalytically produced by eNOS and can prevent CVD. When cells are in oxidative stress or inflammation, NO is catalytically produced by iNOS. Some studies found iNOS expression and NO content of serum in AS mice were higher than those in blank group, while some research found some anti-inflammatory medicine can down-regulate iNOS expression and decrease NO content, thus inhibiting inflammation (26). Hence, these results indicate NO has two sides for AS processing. NO produced by eNOS can protect cells while NO produced by iNOS can promote AS formation. In the model group of this study, eNOS expression decreased while iNOS expression increased and NO content decreased at the same time compared to blank group, indicating PA has a stronger effect on NO produced by eNOS than that by iNOS. Also, after treatment with SB, NO content and iNOS expression significantly decreased compared with model group. This meant the process of NO production also has a connection with p38MAPK/NF-κB signal pathway.

ICAM-1 expression has a close relationship with p38MAPK signal pathway (27). In this study, SB can significantly decrease ICAM mRNA expression, but has no significant influence on ICAM protein expression, indicating SBP regulation of ICAM is only associated with p38MAPK/NF-κB signal pathway at the transcriptional level while its regulation of ICAM after transcription is probably through other ways. Moreover, SB treatment decreased protein and mRNA expression of NF-κB. SB inhibition is similar to SBP inhibition, which indicated SBP regulation of NF-κB expression is also through p38MAPK/NF-κB signal pathway.

SBP regulation on oxidative damaged HUVECs induced by PA is probably achieved by inhibiting p38MAPK/NF-κB signal pathway activity, but this still needs further determination.

## Data Availability Statement

The original contributions presented in the study are included in the article/supplementary materials, further inquiries can be directed to the corresponding author/s.

## Author Contributions

XL: conceptualization. MY: methodology. TY: software. HY, MW, and QP: validation. QP: funding acquisition. All authors have read and agreed to the published version of the manuscript.

## Funding

This research was financially supported by the Science and Technology Project of Xining (No. 2021-Y-15), Beijing Engineering and Technology Research Center of Food Additives, Beijing Technology & Business University (BTBU), and Yulin City Science and Technology Plan Project (No. CXY-2020-074).

## Conflict of Interest

MY, TY, and HY were employed by Puredia Limited. The remaining authors declare that the research was conducted in the absence of any commercial or financial relationships that could be construed as a potential conflict of interest.

## Publisher's Note

All claims expressed in this article are solely those of the authors and do not necessarily represent those of their affiliated organizations, or those of the publisher, the editors and the reviewers. Any product that may be evaluated in this article, or claim that may be made by its manufacturer, is not guaranteed or endorsed by the publisher.

## Abbreviations

SBP: procyanidins from sea-buckthorn
SB203580 (SB): a p38 enzyme inhibitor
CVD: cardiovascular disease
HUVECs: Human umbilical vein endothelial cells
NO: nitric oxide.
